# Virtual Patients Using Large Language Models: Scalable, Contextualized Simulation of Clinician-Patient Dialogue With Feedback

**DOI:** 10.2196/68486

**Published:** 2025-04-04

**Authors:** David A Cook, Joshua Overgaard, V Shane Pankratz, Guilherme Del Fiol, Chris A Aakre

**Affiliations:** 1 Division of General Internal Medicine Mayo Clinic College of Medicine and Science Rochester, MN United States; 2 Multidisciplinary Simulation Center Mayo Clinic College of Medicine and Science Rochester, MN United States; 3 Health Sciences Center University of New Mexico Albuquerque, NM United States; 4 Department of Biomedical Informatics University of Utah School of Medicine Salt Lake City, UT United States

**Keywords:** simulation training, natural language processing, computer-assisted instruction, clinical decision-making, clinical reasoning, machine learning, virtual patient, natural language generation

## Abstract

**Background:**

Virtual patients (VPs) are computer screen–based simulations of patient-clinician encounters. VP use is limited by cost and low scalability.

**Objective:**

We aimed to show that VPs powered by large language models (LLMs) can generate authentic dialogues, accurately represent patient preferences, and provide personalized feedback on clinical performance. We also explored using LLMs to rate the quality of dialogues and feedback.

**Methods:**

We conducted an intrinsic evaluation study rating 60 VP-clinician conversations. We used carefully engineered prompts to direct OpenAI’s generative pretrained transformer (GPT) to emulate a patient and provide feedback. Using 2 outpatient medicine topics (chronic cough diagnosis and diabetes management), each with permutations representing different patient preferences, we created 60 conversations (dialogues plus feedback): 48 with a human clinician and 12 “self-chat” dialogues with GPT role-playing both the VP and clinician. Primary outcomes were dialogue authenticity and feedback quality, rated using novel instruments for which we conducted a validation study collecting evidence of content, internal structure (reproducibility), relations with other variables, and response process. Each conversation was rated by 3 physicians and by GPT. Secondary outcomes included user experience, bias, patient preferences represented in the dialogues, and conversation features that influenced authenticity.

**Results:**

The average cost per conversation was US $0.51 for GPT-4.0-Turbo and US $0.02 for GPT-3.5-Turbo. Mean (SD) conversation ratings, maximum 6, were overall dialogue authenticity 4.7 (0.7), overall user experience 4.9 (0.7), and average feedback quality 4.7 (0.6). For dialogues created using GPT-4.0-Turbo, physician ratings of patient preferences aligned with intended preferences in 20 to 47 of 48 dialogues (42%-98%). Subgroup comparisons revealed higher ratings for dialogues using GPT-4.0-Turbo versus GPT-3.5-Turbo and for human-generated versus self-chat dialogues. Feedback ratings were similar for human-generated versus GPT-generated ratings, whereas authenticity ratings were lower. We did not perceive bias in any conversation. Dialogue features that detracted from authenticity included that GPT was verbose or used atypical vocabulary (93/180, 51.7% of conversations), was overly agreeable (n=56, 31%), repeated the question as part of the response (n=47, 26%), was easily convinced by clinician suggestions (n=35, 19%), or was not disaffected by poor clinician performance (n=32, 18%). For feedback, detractors included excessively positive feedback (n=42, 23%), failure to mention important weaknesses or strengths (n=41, 23%), or factual inaccuracies (n=39, 22%). Regarding validation of dialogue and feedback scores, items were meticulously developed (content evidence), and we confirmed expected relations with other variables (higher ratings for advanced LLMs and human-generated dialogues). Reproducibility was suboptimal, due largely to variation in LLM performance rather than rater idiosyncrasies.

**Conclusions:**

LLM-powered VPs can simulate patient-clinician dialogues, demonstrably represent patient preferences, and provide personalized performance feedback. This approach is scalable, globally accessible, and inexpensive. LLM-generated ratings of feedback quality are similar to human ratings.

## Introduction

Translating advances in biomedical knowledge and knowledge synthesis into data-driven, patient-centered, and contextualized management decisions remains a wicked challenge. As we seek to prevent errors in clinical practice [1,2] and promote high-value care [3,4], we need to better understand clinical reasoning and how to support its development and application [2,5]. Because clinical reasoning is case specific [6] and educationally opportune encounters with real patients are finite, education and research in this field require a scalable approach to emulating authentic patient-clinician interactions. Virtual patients (VPs) powered by large language models (LLMs) offer a potential solution.

VPs—computer screen–based simulations of patient-clinician encounters [7]—have demonstrated efficacy in teaching, assessing, and studying clinical reasoning [8] and could also support validation of decision-support tools before clinical implementation [9,10]. VPs may be particularly important for *management reasoning*, which is a subset of clinical reasoning. In contrast with diagnostic reasoning, management reasoning is arguably more difficult, more complex to study, and more important [11,12]. Yet, it has received scant investigation owing to challenges in replicating management tasks—most notably patient-clinician conversations—which necessarily involve shared decision-making [13-16] and contextualization of care (ie, consideration of social determinants of health, patient preferences, and comorbid conditions) [17-20].

To date, VP use has been limited by the high costs and logistical challenges of large-scale implementation. One survey found that 85% of bespoke VPs cost >US $10,000 per case and required >16 months to produce [21]. Commercial VP libraries exist, but subscriptions are expensive (approximately US $100/student/y). Hence, VP implementations typically comprise few cases and lack case-to-case variability in salient features (eg, diagnosis, illness severity, preferences, and ethnic diversity) [8,21,22].

Providing performance feedback to clinicians is also essential in clinical skill development [23], yet it is commonly of low quality or simply absent [24-27]. Specific, actionable feedback [28-30] on VP-clinician interactions could promote clinical reasoning and communication skills.

LLMs represent a disruptive technology [31], offering an unprecedented opportunity to transform VP production and use, enabling scalable, accessible (ie, inexpensive and low expertise), interoperable, and reusable [32] simulations of patient-clinician encounters. Our aim was to show proof of concept that VPs powered by OpenAI’s generative pretrained transformer (GPT) can generate authentic preference-sensitive dialogues and high-quality feedback. We hypothesized that human ratings of *observed* patient preferences would agree with corresponding *planned* preferences (ie, that GPT would perceptibly represent the intended preference). We compared GPT-4.0-Turbo against the earlier, cheaper GPT-3.5-Turbo, hypothesizing that GPT-4.0-Turbo would be superior. We also piloted GPT to role-play the clinician, hypothesizing that conversations involving human clinicians would be superior.

As a substudy, we aimed to pilot LLMs for rating the quality of VP-clinician dialogues and feedback. Artificial intelligence (AI) has long been used to rate narrative text [33-37], but this typically requires supervised machine learning—using human-graded texts to train the AI system. We explored the use of LLMs without any training exemplars (ie, zero-shot learning).

## Methods

### Overview

**Figure 1:**
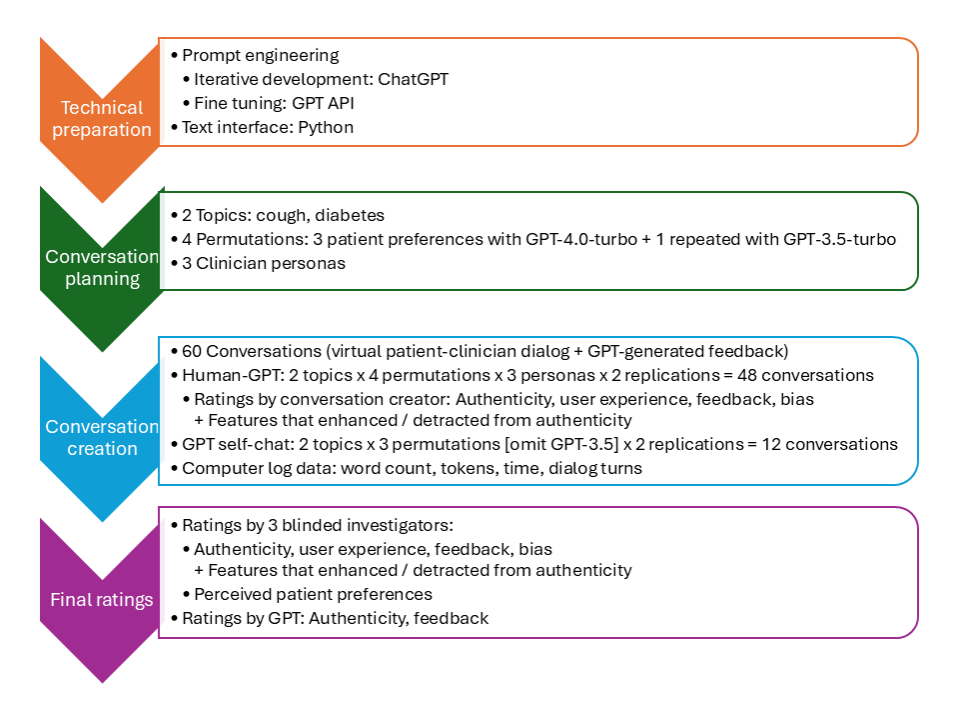
Overview of the study design. GPT=GPT−4.0-turbo except as otherwise noted. A “conversation” refers to the virtual patient–clinician dialogue plus feedback. API: application programming interface; GPT: generative pretrained transformer.

We conducted an intrinsic evaluation study (ie, a study that evaluates the quality of computer-generated outputs on specific predefined tasks, rather than real-world learners or tasks), rating the quality of 60 conversations (ie, the combination of VP-clinician dialogue and LLM-generated performance feedback) between an LLM-powered VP and a clinician. We created 3 novel instruments to rate dialogue authenticity and feedback quality. Three physicians and GPT rated all conversations. Figure 1 summarizes the study design.

### Ethical Considerations

No human subjects were involved in this study, other than the study investigators. As such, we did not pursue appraisal by an ethics review board.

### Technical Preparation: LLM-Powered VP Interface

We used Python to create a text VP interface, as previously described [38], that accesses GPT through the OpenAI application programming interface (API). We iteratively and rigorously engineered detailed “prompts” guiding GPT to emulate a diagnosis-focused or management-focused VP and provide feedback. To instantiate a specific VP, the interface accesses a 1-page case description. Narrative S1 in Multimedia Appendix 1 reports the full prompt and 1 case description.

### Conversation Planning

We selected as topics 2 common problems in ambulatory medicine: chronic cough (a diagnostic task) and diabetes (a management task). For each topic, we created a written description of a prototypical scenario. In this pilot study we did not base scenarios on specific real patients.

We planned 4 permutations per topic by varying the patient preferences or GPT model:

- Case 1: patient has good insurance and wants to avoid tests or new medications (GPT-4.0-Turbo)
- Case 2: patient has financial concerns such as limited income and poor insurance (GPT-4.0-Turbo)
- Case 3: patient is anxious and pushes for more tests and more aggressive treatments (GPT-4.0-Turbo)
- Case 4: same as case 1 (GPT-3.5-Turbo)

The details on dialogue permutations are provided in Table S1 in Multimedia Appendix 1. The dialogues were further permuted for 3 clinician personas: an average third-year medical student, a poor-performing third-year medical student, and an average second-year internal medicine resident.

### Conversation Creation

We used the LLM-powered VP interface to create 48 simulated conversations between the VP and a human clinician. A representative conversation is provided in Narrative S2 in Multimedia Appendix 1. A board-certified internal medicine physician role-played the clinician twice for each permutation (ie, 2 topics, 4 case variations, 3 clinician personas, and 2 replications=48 conversations). One investigator role-played all conversations for cough and another investigator role-played those for diabetes. The investigator knew which clinician persona to portray but was not told which case variation GPT portrayed. Using the instruments defined later in this report, the investigator rated dialogue quality immediately after ending each dialog. GPT (via the VP interface) then offered detailed performance feedback, and the investigator rated feedback quality and perceived bias.

In addition, we used GPT-4.0-Turbo to play the role of an “excellent physician,” and “self-chat” as both the VP and clinician using independent GPT threads for cases 1 to 3, with 2 replications each (ie, 2 topics, 3 case variations, and 2 replications=12 GPT-GPT self-chats).

Each conversation was saved verbatim, along with time spent, word count, and GPT “tokens” used. We calculated costs using GPT pricing.

### Instrument Creation

#### Overview

We created 3 novel instruments for rating the quality of VP dialogues and feedback (Table 1), and 1 item to flag potential bias. We also collected granular information on conversation features that influenced authenticity. For the 3 novel instruments, we conducted a validation study collecting validity evidence from 4 of 5 potential sources [39,40]: content (ie, grounding of the instruments in theory and prior empirical work); internal structure (ie, rating reproducibility); relations with other variables (ie, sensitivity of ratings to case differences, including expectation of higher ratings for more advanced LLM models and human clinician personas); and response process (ie, clarification on why raters responded as they did). Narrative S3 in Multimedia Appendix 1 further describes instrument development and validation planning.

**Table 1:** Rating scales for appraising conversation quality: constructs, items, operational clarifications, and reproducibility^a^.

Item		Verbatim item wording	Operational clarifications^a^	ICC^b^: human (N=3)^c^	ICC: GPT^d^ (N=3)^c^
**Dialogue authenticity**					
	Humanlike	The virtual patient’s responses were humanlike.	Sensible, natural, and conversational; uses appropriate word choice, phrasing, and tone	0.34	0.29
	Coherent	The virtual patient’s responses were coherent.	Contextually appropriate and internally consistent (ie, logical) over the course of the dialogue	0.40	0.45
	Personal	The virtual patient’s responses were personal.	Reflecting preferences, opinions, values, and priorities; not overly agreeable or pleasing	0.22	0.35
	Relevant	The virtual patient’s responses were relevant and meaningful.	Meaningful, useful, helpful as a clinically relevant simulation; requires or supports clinical reasoning; stimulates appropriate emotions and empathy	0.30	0.20
	Overall	The dialogue as a whole mirrored a real-life patient-clinician conversation.	—^e^	0.34	0.49
**User experience**					
	Realness	This was an authentic representation of a real-world experience.	Similar to a real-world situation	0.37	0.29
	Cognitive authenticity	I had to continuously revise my mental image of the problem using new information.	Requires or stimulates the same mental activities, same decisions as in real situation; real professional demand	0.24	—
	Variability	The interaction seemed unscripted and appropriately complex.	Reflects natural variation in responses; spontaneous, unstructured, unplanned, and flexible; complex, multidimensional (not superficial); not robot-like or prefabricated	0.19	—
	Involvement^f^	I was fully engaged in this conversation.	Immersed, focused (not distracted), captivated; stimulated empathy and authentic emotions	X^g^	—
	Overall	I felt as if I were the doctor.	—	0.17	—
**Feedback**					
	Evidence based	The feedback correctly identifies important weaknesses and strengths in the clinician’s performance.	Specific observations of behavior; accurately interpreted; well prioritized	0.15	0.09
	Actionable	The feedback contains suggestions that are specific and actionable.	Specific and actionable suggestions for behavior change	0.17	0.26
	Connected	The feedback correctly connects each suggestion with specific strengths and weaknesses.	Explicit and logical connection between the observed behaviors and suggested changes	0.22	0.25
	Balanced	The feedback balances corrective and reinforcing statements appropriate to the clinician’s performance.	Includes both praise and critique; a balance of positive and negative statements matches actual performance	0.08	0.16
**Bias (overall)**		Did you detect any indication of bias or stereotyping in the dialogue or feedback?	Includes stereotyping, disparagement, dehumanization, erasure, and inequitable performance	1^f^	—

^a^All conversations were rated at the time of their creation by the physician who created them (“initial ratings”) and later by blinded human raters and by GPT (“final ratings”). Items were presented in the sequence shown above. Operational clarifications were included only for final ratings. A “conversation” refers to the VP-clinician dialogue plus feedback. During conversation creation, each dialogue was rated before feedback was provided. Response options for all rating scale items ranged from 1=strongly disagree to 6=strongly agree. For authenticity and experience, a rating of 6 was operationally defined as “This is exactly what I would expect in a real conversation; this could have come from a human patient.” For feedback, a rating of 6 was operationally defined as “This is surprisingly good, better than I would expect from a trained human clinician-supervisor.” See Box S1 in Multimedia Appendix 1 for additional details on operational criteria. Response options for bias were Yes and No.

^b^ICC: intraclass correlation coefficient.

^c^An ICC representing the overall reproducibility coefficient for a single rating. “Human” indicates agreement across 3 blinded board-certified internal medicine physicians; “GPT” indicates agreement across 3 rating runs from GPT-4.0-Turbo.

^d^GPT: generative pretrained transformer.

^e^GPT did not rate user experience and bias.

^f^This item was created as part of our instrument, reflecting the corresponding domain in the underlying conceptual framework. However, we did not code this feature in this study, as we investigators did not feel authentically “engaged” in the task when creating multiple conversations. This item could be used in future studies with real learners.

^g^There was 100% agreement across all raters on the bias item.

#### Dialogue Rating Items

Two instruments focused on the dialogues: dialogue authenticity and user (ie, clinician) experience. To generate items to rate dialogue authenticity, we drew on the literature on dialogue systems and natural language generation [41-51] from which we distilled 5 repeatedly emphasized constructs: responses are *humanlike* (ie, sensible, natural, and avoiding bias), *coherent* (ie, contextually appropriate and internally consistent), engaging or *personal* (ie, reflecting preferences, empathy, and personality), helpful or *relevant* (ie, specific, useful, and meaningful), and *correct* (for knowledge-delivery systems). We dropped "correct" since our purpose was dialogue and not knowledge delivery. We considered but omitted a domain for fluency because recent literature suggests that fluency can be presumed for contemporary AI models [42,44,46]. We created 1 item for each construct and an overall item, resulting in a 5-item instrument.

To generate items to rate user experience, we merged 2 conceptual frameworks for measuring authenticity in VPs—one emphasizing decision-making and cognitive strategies [52] and the other highlighting realism, empathy, and variability [22,53]. We added a third empirically derived framework for evaluating “presence” in virtual reality (ie, realness, involvement, and spatial “physical” presence) [54,55]. We synthesized these into 4 constructs: *realness* (ie, similar to a real-world situation); *cognitive authenticity* (ie, real mental activities and decisions); *variability* (ie, case-to-case variation and spontaneous responses); and *involvement* (ie, user engaged and immersed). We created 1 item for each construct and an overall item, resulting in a 5-item instrument. In this study, we did not rate “involvement” because we never felt “immersed” when creating and rating multiple conversations; however, we plan to rate this in future studies.

#### Feedback Items

To generate items to rate feedback, we integrated findings from focus group studies [24,28], published instruments [30,56,57], and other empirical and conceptual studies [29,58-61] and identified 4 recurrent constructs: *evidence-based* (ie, behavior-focused) observations; specific, *actionable* suggestions; observations explicitly *connected* with suggestions; and *balanced* praise and critique. We created 1 item for each construct, resulting in a 4-item instrument. We did not rate feedback “overall”; instead, we calculated the average rating.

#### Further Procedures for Dialogue and Feedback Instruments

Three experts in VPs or natural language generation reviewed the 3 instruments and approved them with minor clarifications. Response options ranged from 1=strongly disagree to 6=strongly agree. After case creation and before the final rating phase, we added brief operational criteria for each response option (Box S1 in Multimedia Appendix 1).

#### Bias Item

Bias—“skew that produces a type of harm toward different social groups” [62]—is a well-known risk in AI generally and natural language generation specifically [62-65]. Bias can arise from the input (ie, training) data, annotation process, input representations, models, or research design [63], resulting in harms of stereotyping, disparagement, dehumanization, erasure, and inequitable performance [62] to nondominant groups. These groups can be defined by demographics such as gender, age, gender orientation, physical appearance, disability, nationality, ethnicity, race, socioeconomic status, religion, and culture [64]. Raters were instructed to flag and describe any bias or stereotyping in the dialogue or feedback, specifically considering the sources and groups noted earlier.

#### Conversation Features That Influenced Authenticity

Following the dialogue ratings, and again after the feedback ratings, we asked, “What specific features of this [dialog | feedback] detracted from its authenticity?” and “What specific features enhanced its authenticity?” Investigators responded using free text during conversation creation. We collated responses into a list of features and selected from this list during the final ratings.

### Final Ratings of Conversations

As described earlier, each investigator rated conversation quality at the time of conversation creation.

Later, all conversations were rated again by all 3 investigators for dialogue authenticity, user experience, feedback quality, and bias (ie, “final ratings”). At this stage, raters also indicated their perception of patient preferences represented in the dialogue regarding (1) less versus more testing, (2) the importance of cost, and (3) prioritization of lifestyle or control of illness. They also indicated specific features of the conversation that detracted from or enhanced its authenticity.

Raters were blinded to the permutation. Conversations were randomized for final ratings (ie, a unique sequence for each rater). Raters entered data using an internet-based form implemented using DistillerSR.

We also used GPT-4.0-Turbo (via the OpenAI API) to rate each conversation 3 times for dialogue authenticity and feedback quality but not user experience.

### Data Analysis

#### Reproducibility of Final Ratings

To appraise rating reproducibility, we estimated variance components and calculated a single-rating intraclass correlation coefficient (ICC), which was interpreted using criteria from Landis and Koch [66] (ie, 0-0.2=slight; 0.21-0.4=fair; 0.41-0.6=moderate; and 0.61-0.8=substantial).

#### Comparison Across Design Features

We selected 5 outcomes (ie, overall authenticity, humanlike, overall experience, realness, and average feedback) as most aligned with our study aims and compared these across GPT models, topics, clinician personas, and human versus LLM raters. Using mixed models ANOVA, we conducted paired analyses that accounted for features of the factorial design and, for final ratings, repeated measures from multiple raters. We used SAS 9.4 (SAS Institute Inc) for all analyses and set the α level at .05. We make inferences of statistical significance using 95% CIs.

## Results

### Instrument Validation

We conducted a validation study for the novel instruments for rating dialogue authenticity, user experience, and feedback quality. Evidence for content is presented in the Methods section and Narrative S3 in Multimedia Appendix 1. Additional evidence is presented and discussed subsequently, including evidence for internal structure (ie, rating reproducibility was suboptimal), relations with other variables (ie, ratings differed as expected across conversation subgroups), and response process (ie, questions probed investigators’ thought processes regarding features that detracted from or enhanced conversation quality).

### Conversation Creation Resources

We created 48 VP-clinician conversations (ie, dialogue plus feedback) with human physicians playing the clinician role and 12 conversations with GPT as the clinician. Each human-created conversation lasted for an average of 622 seconds (of which GPT’s responses took 90 seconds) and cost US $0.50 (see Table 2 for additional details including estimates of measurement variability, ie, SD).

GPT-3.5-Turbo was significantly faster than GPT-4.0-Turbo (62 vs 100 seconds; difference 38, 95% CI 29-47) and much cheaper (US $0.02 vs US $0.51 per conversation), although quality was substantially lower (see the subsequent section). Compared with diabetes, cough conversations required substantially more GPT time (122 vs 59 seconds) and tokens (72,745 vs 27,241) even though the dialogue itself was only slightly longer (1165 vs 908 words). This was due to more back-and-forth turns in the dialogue (mean 37 vs 14 turns), because each time GPT processes a clinician statement (eg, even a short query like “Do you have heartburn?”), the entire dialogue is resubmitted to GPT as context.

The average time for the 12 GPT-GPT (ie, self-chat) conversations was 113 seconds: 62 seconds for the clinician, and 51 seconds for the VP. The average cost was US $0.29 because these dialogues had fewer turns (mean 21 turns).

**Table 2:** Conversation creation: resource metrics and initial ratings of conversation quality^a^.

Metric		Human clinician, mean (SD), median						Self-chat (all, n=12), mean (SD), median
		All (n=48)	GPT-4.0 (n=36)	GPT-3.5 (n=12)	Diabetes (n=24)	Cough (n=24)		
**Resources and time**								
	Total time (s)^b^	622 (173), 611	653 (168), 669	551 (171), 508	617 (158), 611	627 (189), 619	113 (20), 107	
	Physician time (s)^b^	534 (166), 553	553 (162), 556	488 (173), 477	562 (151), 553	510 (178), 511	62 (14), 57	
	Virtual patient (GPT) time (s)	90 (38), 76	100 (36), 99	62 (28), 63	59 (16), 65	122 (24), 129	51 (8), 51	
	Words (dialogue)^c^	1037 (302), 1003	1092 (304), 1059	871 (238), 810	908 (232), 942	1165 (313), 1165	1377 (351), 1291	
	Words (feedback)^c^	387 (118), 425	449 (50), 450	202 (38), 198	371 (94), 413	403 (138), 458	424 (43), 407	
	Tokens (total)^c^	49,993 (25,609), 47,621	50,788 (25,788), 46,826	47,607 (26,036), 47,894	27,241 (6139), 26,205	72,745 (14,904), 66,960	28,628 (7997), 27,209	
	Dialogue turns^c^	26 (13), 24	26 (13), 24	26 (13), 24	14 (3), 15	37 (7), 34	21 (6), 20	
	Cost per conversation, US $^d^	0.50 (0.26), 0.48	0.51 (0.26), 0.47	0.02 (0.01), 0.02	0.27 (0.06), 0.26	0.73 (0.15), 0.67	0.29 (0.08), 0.27	
**Dialogue authenticity^e^**								
	Overall	4.6 (0.6), 5	4.8 (0.6), 5	3.9 (0.3), 4	4.5 (0.6), 4.5	4.8 (0.7), 5	—^f^	
	Humanlike	4.8 (0.7), 5	5.1 (0.5), 5	3.9 (0.5), 4	4.7 (0.6), 5	4.9 (0.8), 5	—	
	Coherent	5.4 (0.6), 5	5.5 (0.6), 5.5	5.3 (0.7), 5	4.9 (0.3), 5	6.0 (0.2), 6	—	
	Personal	5.0 (0.7), 5	5.4 (0.5), 5	4.1 (0.3), 4	4.8 (0.4), 5	5.3 (0.8), 6	—	
	Relevant	5.3 (0.7), 5	5.4 (0.6), 5	4.7 (0.7), 5	4.9 (0.4), 5	5.6 (0.6), 6	—	
**User experience^e^**								
	Overall	4.9 (0.6), 5	5.0 (0.5), 5	4.4 (0.5), 4	4.7 (0.5), 5	5.0 (0.6), 5	—	
	Realness	4.6 (0.7), 5	4.8 (0.7), 5	4.0 (0.4), 4	4.3 (0.8), 5	4.8 (0.6), 5	—	
	Cognitive authenticity	4.5 (0.8), 4	4.6 (0.8), 5	4.2 (0.7), 4	3.9 (0.4), 4	5.1 (0.5), 5	—	
	Variability	5.0 (0.7), 5	5.2 (0.6), 5	4.5 (0.7), 5	4.8 (0.4), 5	5.3 (0.8), 5	—	
**Feedback^e^**								
	Average	4.6 (0.9), 5	4.9 (0.6), 5	3.7 (1.0), 4	4.4 (0.9), 5	4.9 (0.8), 4.6	—	
	Evidence based	4.3 (1.1), 4.5	4.6 (0.9), 5	3.5 (1.0), 3.5	4.4 (1.0), 5	4.3 (1.1), 4	—	
	Actionable	4.9 (0.8), 5	5.2 (0.5), 5	4.0 (1.0), 4	4.6 (0.8), 5	5.3 (0.7), 5	—	
	Connected	4.8 (0.9), 5	5.1 (0.6), 5	3.8 (0.9), 4	4.5 (0.8), 5	5.1 (0.9), 5	—	
	Balanced	4.5 (1.1), 5	4.8 (0.9), 5	3.6 (1.3), 4	4.2 (1.2), 5	4.8 (0.9), 5	—	

^a^The clinician was a human physician for the “human clinician” conversations and GPT-4.0-Turbo for the “self-chat” conversations. The virtual patient was GPT for all conversations.

^b^n=37 for total time and human physician time, after excluding 11 conversations in which the recorded time was inexact due to interruptions.

^c^Dialogue was generated as an interaction between the virtual patient (GPT) and clinician (human or GPT). Feedback was generated by GPT. A “conversation” refers to the VP-clinician dialogue plus feedback. Tokens include entire conversation (both dialogue and feedback; and for self-chat, both patient and physician).

^d^Pricing (per OpenAI, May 30, 2024): US $1.00/100,000 tokens for GPT-4.0-Turbo; US $0.05/100,000 tokens for GPT-3.5-Turbo.

^e^All conversations (dialogue and feedback) were rated at the time of their creation by the physician who created them, immediately following the dialogue and feedback (GPT did not provide initial ratings following self-chat). Response options for all items ranged from 1=strongly disagree to 6=strongly agree.

^f^Not applicable.

### Representation of Patient Preferences

Each case was written to represent patient preferences in testing or treatment, cost of care, and prioritization of illness control versus lifestyle. During the blinded final rating, we independently indicated whether the VP represented such preferences in the dialogue. The reproducibilities (ie, ICCs) for these ratings were as follows: testing or treatment, 0.59; cost of care, 0.75; and prioritization of control, 0.39.

VPs demonstrably represented planned preferences with high frequency (Table 3). For dialogues created using GPT-4.0-Turbo, 5 of 6 nonneutral planned preferences were recognized as such in ≥54% of dialogues, and all 3 neutral planned preferences were rated as “no opinion” in ≥90% of the dialogues. We observed comparable results for GPT-3.5-Turbo.

**Table 3:** Patient preferences reflected in dialogues: planned versus perceived by raters.

Perceived preference (human rating)		Case 1 (n=48^a^), n (%)		Case 2 (n=48^a^), n (%)		Case 3 (n=48^a^), n (%)		Case 1 GPT-3.5 (n=36)^a^, n (%)		Diabetes (n=90), n (%)		Cough (n=90), n (%)	
**Testing or treatment**													
	Less		*20 (42)* ^b^		*35 (73)*		0 (0)		*17 (47)*		41 (46)		31 (34)
	No opinion		27 (56)		13 (27)		11 (23)		17 (47)		25 (28)		43 (48)
	More		1 (2)		0 (0)		*37 (77)*		2 (6)		24 (27)		16 (18)
**Cost**													
	Lower		3 (6)		*47 (98)*		1 (2)		2 (6)		25 (28)		28 (31)
	No opinion		*43 (90)*		1 (2)		21 (44)		*29 (81)*		39 (43)		55 (61)
	Not an issue		2 (4)		0 (0)		*26 (54)*		5 (14)		26 (29)		7 (8)
**Impact on life**													
	Prioritize lifestyle		3 (6)		3 (6)		0 (0)		1 (3)		6 (7)		1 (1)
	No opinion		*45 (94)*		*43 (90)*		19 (40)		*34 (94)*		65 (72)		76 (84)
	Prioritize illness control		0 (0)		2 (4)		*29 (60)*		1 (3)		19 (21)		13 (14)

^a^This table indicates patient preferences as planned and prompted in the case description provided to the generative pretrained transformer (GPT), and preferences as perceived by blinded human raters to be represented in the dialogues. Case 1 was planned to reflect desire for less testing or treatment. Case 2 was planned to reflect strong desire for lower cost, and hence less testing or treatment. Case 3 was planned to reflect desire for more testing or treatment, cost not an issue, and prioritization of illness control over lifestyle. See Table S1 in Multimedia Appendix 1 for details on planned case features.

^b^Italicized values indicate dialogues in which prompted and perceived preferences align.

### Conversation Quality: Authenticity, Experience, and Feedback

Conversation quality was appraised by 1 rater at the time of creation and later by all 3 investigators (final ratings).

#### Conversation Creation

During creation, mean dialogue ratings ranged from 4.8 to 5.4 (out of a maximum rating of 6) for authenticity and from 4.5 to 5.0 for user experience (Table 2). Feedback quality ranged from 4.3 to 4.9. Ratings were significantly higher for GPT-4.0-Turbo versus GPT-3.5-Turbo (difference: dialogue overall 0.92, 95% CI 0.64-1.19; experience overall 0.58, 95% CI 0.21-0.96; feedback average 1.33, 95% CI 0.80-1.87).

#### Final Ratings

The reproducibilities of authenticity and experience final ratings were typically “fair,” with ICCs ranging from 0.17 to 0.40 (Table 1). In contrast, reproducibilities for feedback ratings were “slight,” with all but 1 domain ≤0.17. We examined the variance components (Tables S2 and S3 in Multimedia Appendix 1) and found very small between-rater variances (representing ≤5% of total variance for all except for feedback evidence based, which was 18%). In contrast, we found large (≥60% of total) between-replication variances, which reflect a combination of true differences in GPT performances and within-rater variability.

Mean final ratings ranged from 4.6 to 5.0 for authenticity, 4.6 to 4.9 for experience, and 4.5 to 4.9 for feedback (see Table 4 and Table S4 in Multimedia Appendix 1 for details, including estimates of measurement variability and subgroup analyses).

**Table 4:** Final ratings of conversation quality: mean and median scores^a^.

			Rater, mean (SD), median											Case, mean (SD), median						
			All human raters (N=180)		GPT^b^ rater (N=180)		Human rater 1 (N=60)		Human rater 2 (N=60)		Human rater 3 (N=60)		Case 1 (N=48)			Case 2 (N=48)		Case 3 (N=48)		Case 1, GPT-3.5^c^ (N=36)
**Dialogue authenticity**																				
	Overall	4.7 (0.7), 5		5.2 (0.6), 5		4.8 (0.8), 5		4.8 (0.6), 5		4.6 (0.7), 5		4.7 (0.7), 5			4.9 (0.8), 5		4.8 (0.6), 5		4.4 (0.8), 5	
	Humanlike	4.6 (0.8), 5		5.6 (0.5), 6		4.8 (0.9), 5		4.6 (0.5), 5		4.5 (0.8), 5		4.5 (0.7), 5			5.0 (0.7), 5		4.8 (0.6), 5		4.1 (0.9), 4	
	Coherent	5.0 (0.6), 5		5.6 (0.5), 6		5.0 (0.8), 5		4.9 (0.5), 5		5.0 (0.6), 5		5.0 (0.5), 5			5.1 (0.5), 5		5.2 (0.4), 5		4.4 (0.9), 5	
	Personal	5.0 (0.6), 5		5.1 (0.6), 5		5.2 (0.9), 5		5.0 (0.2), 5		4.9 (0.7), 5		5.0 (0.5), 5			5.2 (0.6), 5		5.1 (0.7), 5		4.6 (0.6), 5	
	Relevant	4.9 (0.6), 5		5.8 (0.4), 6		4.8 (0.9), 5		4.9 (0.4), 5		4.9 (0.5), 5		4.9 (0.5), 5			5.0 (0.7), 5		5.0 (0.5), 5		4.6 (0.8), 5	
**User experience**																				
	Overall	4.9 (0.7), 5		—^d^		4.9 (1.0), 5		4.9 (0.3), 5		4.8 (0.6), 5		4.7 (0.7), 5			5.1 (0.7), 5		4.9 (0.7), 5		4.8 (0.6), 5	
	Realness	4.6 (0.8), 5		—		4.7 (1.1), 5		4.6 (0.6), 5		4.5 (0.8), 5		4.6 (0.7), 5			4.9 (0.8), 5		4.8 (0.8), 5		4.1 (0.9), 4	
	Cognitive authenticity	4.8 (0.7), 5		—		5.0 (0.9), 5		4.9 (0.3), 5		4.6 (0.7), 5		4.8 (0.7), 5			5.0 (0.7), 5		4.9 (0.8), 5		4.7 (0.5), 5	
	Variability	4.8 (0.9), 5		—		4.6 (1.2), 5		4.9 (0.3), 5		4.7 (0.8), 5		4.6 (0.9), 5			5.0 (0.8), 5		4.8 (0.9), 5		4.5 (0.9), 5	
**Feedback**																				
	Average	4.7 (0.6), 4.9		4.6 (0.2), 4.8		4.8 (0.8), 4.9		4.8 (0.4), 5		4.5 (0.6), 4.8		4.8 (0.5), 5			4.9 (0.6), 5		4.8 (0.5), 5		4.1 (0.7), 4.1	
	Evidence based	4.5 (0.9), 5		4.9 (0.3), 5		4.7 (1.0), 5		4.8 (0.4), 5		4.1 (0.9), 4		4.7 (0.7), 5			4.7 (0.8), 5		4.6 (0.8), 5		3.9 (1.1), 4	
	Actionable	4.9 (0.6), 5		4.5 (0.5), 5		5.1 (0.8), 5		4.9 (0.3), 5		4.8 (0.6), 5		5.0 (0.5), 5			5.1 (0.5), 5		5.1 (0.5), 5		4.4 (0.7), 5	
	Connected	4.9 (0.7), 5		4.6 (0.5), 5		4.9 (0.9), 5		4.9 (0.4), 5		4.8 (0.6), 5		5.1 (0.5), 5			5.0 (0.7), 5		5.1 (0.4), 5		4.2 (0.8), 4	
	Balanced	4.5 (0.9), 5		4.5 (0.6), 5		4.5 (1.1), 5		4.6 (0.7), 5		4.4 (0.9), 5		4.6 (0.9), 5			4.7 (0.8), 5		4.6 (0.9), 5		4.0 (0.9), 4	

^a^All conversations (ie, dialogue and feedback) were rated for “final ratings” by 3 blinded human raters (ie, board-certified internal medicine physicians) and by GPT. Results are reported as unweighted mean (SD) and median across all conversations. A “conversation” refers to the VP-clinician dialogue plus feedback. Response options for all items ranged from 1=strongly disagree to 6=strongly agree. Table S4 in Multimedia Appendix 1 reports additional rating subgroups (ie, by topic and clinician persona).

^b^GPT: generative pretrained transformer.

^c^“GPT-3.5” conversations used GPT-3.5-Turbo as the virtual patient (N=36 because these did not include 12 self-chat conversations). All other conversations used GPT-4.0-Turbo.

^d^GPT did not rate user experience.

We report final ratings subgroup comparisons in Table 5. Differences between topics were small. All ratings were higher for GPT-4.0-Turbo versus GPT-3.5-Turbo (ie, differences ranging from 0.17 to 0.71), although differences did not always reach statistical significance (as indicated by the 95% CIs). Conversations involving human clinicians had higher experience ratings than those with GPT as clinician (ie, differences ≥0.57) but similar authenticity (ie, differences ≤0.31) and—as would be expected—similar feedback ratings (ie, difference −0.05). Among human clinicians, the resident persona had higher ratings than the poor medical student, and these differences (≥0.48) were statistically significant for authenticity and experience. No instances of potential bias were identified during creation or final rating.

**Table 5:** Final ratings of conversation quality: subgroup comparisons^a^.

Outcome	Topic: Diabetes vs cough (n=180), mean difference (95% CI)	GPT^b^ model: 4.0 vs 3.5 (case 1; n=72), mean difference (95% CI)	Clinician: human vs GPT (n=180), mean difference (95% CI)	Clinician: resident vs medical student persona (n=144), mean difference (95% CI)^c^	Rater: human vs GPT (n=360), mean difference (95% CI)
Dialogue authenticity: overall	0.14 (−0.25 to 0.54)	0.42 (−0.19 to 1.02)	0.31 (−0.25 to 0.88)	0.69 (0.26 to 1.12)	−0.52 (−0.85 to −0.19)
Dialogue authenticity: humanlike^d^	0.09 (−0.32 to 0.50)	0.50 (−0.16 to 1.16)	0.12 (−0.46 to 0.70)	0.71 (0.22 to 1.20)	−0.98 (−1.24 to −0.71)
User experience: overall	0.03 (−0.37 to 0.44)	0.17 (−0.39 to 0.72)	0.57 (0.04 to 1.11)	0.48 (0.07 to 0.88)	—^e^
User experience: realness^d^	0.18 (−0.28 to 0.63)	0.58 (−0.08 to 1.25)	0.69 (0.06 to 1.33)	0.75 (0.24 to 1.26)	—^e^
Feedback: average	0.03 (−0.33 to 0.38)	0.71 (0.13 to 1.28)	−0.05 (−0.51 to 0.41)	0.17 (−0.24 to 0.59)	0.10 (−0.37 to 0.58)

^a^All conversations (dialogue and feedback) were rated for “final ratings” by 3 blinded human raters (ie, board-certified internal medicine physicians) and by GPT. Results reported here reflect adjusted mean differences between groups accounting for repeated measures on conversations and Tukey-adjusted 95% CI. A “conversation” refers to the VP-clinician dialogue plus feedback. Conversations included in each analysis were matched according to design features; nonmatching conversations were excluded. Response options for all items ranged from 1=strongly disagree to 6=strongly agree.

^b^GPT: generative pretrained transformer.

^c^This contrast was selected for reporting post hoc, after the omnibus test across all human clinician personas revealed statistically significant differences (*P*≤.03) for all outcomes except feedback. None of the other pairwise contrasts among human-played personas reached statistical significance.

^d^These outcomes were selected a priori for reporting because they closely aligned with the overarching study aim.

^e^GPT did not rate user experience.

### Features That Detracted From or Enhanced Authenticity

We identified features that detracted from or enhanced conversation authenticity (Table 6). Across 180 dialogues, the most frequent detractors were that GPT was verbose or used atypical vocabulary (93/180, 51.6%), was overly agreeable (56/180, 31.1%), repeated the question as part of the response (47/180, 26.1%), was too easily convinced by clinician suggestions (35/180, 19.4%), or was not offended or confused by poor clinician performance (eg, jargon and poorly worded questions; 32/180, 17.8%). Enhancers included expressing an explicit preference or choice (ie, especially preferences contrary to the clinician’s initial suggestion, 106/180, 58.9%), expressing appropriate emotion (38/180, 21.1%), and notably natural speech (38/180, 21.1%).

**Table 6:** Features that detracted from or enhanced virtual patient conversations.

Feature^a^				All (n=180), n (%)		Diabetes (n=90), n (%)		Cough (n=90), n (%)
**Dialogue**								
	**Detracted**							
		Responses reflect atypical word choice, verbose	93 (51.7)		50 (55.6)		43 (47.8)	
		Overly agreeable	56 (31.1)		35 (38.9)		21 (23.3)	
		Repeated question as part of response	47 (26.1)		16 (17.8)		31 (34.4)	
		Easily convinced or manipulated by clinician	35 (19.4)		23 (25.6)		12 (13.3)	
		Not offended or confused by poor clinician performance (including jargon)	32 (17.8)		20 (22.2)		12 (13.3)	
		Clinician dialogue was unrealistic	29 (16.1)		14 (15.6)		15 (16.7)	
		Volunteered too much information (without being asked)	28 (15.6)		15 (16.7)		13 (14.4)	
		Test ordering and reporting was unrealistic	23 (12.8)		1 (1.1)		22 (24.4)	
		Responses did not make sense	12 (6.7)		2 (2.2)		10 (11.1)	
		Offered excessive teaching support	10 (5.6)		4 (4.4)		6 (6.7)	
		Switched to playing role of doctor	6 (3.3)		0 (0)		6 (6.7)	
	**Enhanced**							
		Expressed preference, challenged recommendations, made clear choice	106 (58.9)		57 (63.3)		49 (54.4)	
		Expressed appropriate emotion	40 (22.2)		23 (25.6)		17 (18.9)	
		Very natural flow; authentic word choice; fluent	38 (21.1)		24 (26.7)		14 (15.6)	
		Challenged clinician when vague or nonsensical	31 (17.2)		6 (6.7)		25 (27.8)	
**Feedback**								
	**Detracted**							
		Too positive or insufficient critique (relative to actual performance)	42 (23.3)		17 (18.9)		25 (27.8)	
		Omission: behavioral weakness or strength not mentioned	41 (22.8)		18 (20)		23 (25.6)	
		Inaccurate: “Omitted” behaviors really *were* done	39 (21.7)		19 (21.1)		20 (22.2)	
		Inaccurate: “Needed” behaviors really not needed	32 (17.8)		19 (21.1)		13 (14.4)	
		Too long, unrealistically detailed	24 (13.3)		9 (10)		15 (16.7)	
		Too negative or insufficient praise (relative to actual performance)	23 (12.8)		13 (14.4)		10 (11.1)	
		Inaccurate: “Observed” behaviors really not done	22 (12.2)		15 (16.7)		7 (7.8)	
		Too vague, brief	19 (10.6)		11 (12.2)		8 (8.9)	
		Omission: inappropriate treatment plan not mentioned	17 (9.4)		9 (10)		8 (8.9)	
		Inaccurate: a suggested clinical test or treatment not really needed	15 (8.3)		10 (11.1)		5 (5.6)	
	**Enhanced**							
		Notably specific, actionable, constructive, accurate	75 (41.7)		41 (45.6)		34 (37.8)	
		Suggested notably useful clinical action	63 (35)		31 (34.4)		32 (35.6)	
		Identified notably or subtly good or bad behavior	46 (25.6)		22 (24.4)		24 (26.7)	
		Notably well justified or prioritized	31 (17.2)		14 (15.6)		17 (18.9)	
		Notably balanced; limited praise for poor performance	12 (6.7)		3 (3.3)		9 (10)	

^a^We inductively iteratively developed a list of detracting and enhancing features throughout the process of conversation creation and final ratings, and each rater then independently marked the presence of each feature as it was noted.

For feedback, detractors included excessively positive feedback relative to actual performance (42/180, 23.3%), failure to mention an important weakness or strength (41/180, 22.8%), inaccuracies due to claimed omissions that were actually done (39/180, 21.7%), or suggested behaviors that were not really needed (32/180, 17.8%). Enhancers included being notably specific or actionable (75/180, 41.7%), suggesting a useful clinical action (63/180, 35%), and recognizing a subtle aspect of clinician performance (46/180, 25.5%).

### Human Versus LLM Quality Ratings

We used GPT-4.0-Turbo to rate each conversation 3 times, requiring 121,860 tokens (US $1.22) per run. GPT took 228 to 506 seconds to rate authenticity and 221 to 234 seconds to rate feedback for all conversations. In contrast with human ratings, between-replication variance in ratings approached 0, such that all nonfeature variance resulted from run-to-run inconsistencies in GPT ratings (Table S3 in Multimedia Appendix 1). The resulting ICCs (Table 1) were on par with those of human raters.

In paired (ie. feature-matched) analyses, authenticity ratings (Table 4) were significantly lower (Table 5) for human-generated versus GPT-generated ratings (ie, −0.98 points for humanlike; −0.52 points overall), whereas feedback ratings were similar for both (ie, only 0.10 points higher).

## Discussion

### Principal Findings

This study explored 4 applications of LLMs for clinical education: a low-cost, scalable LLM-powered interactive VP; LLM-generated feedback on clinician performance; LLM role-playing the clinician; and LLM-generated ratings of dialogue and feedback. This is the first study to empirically evaluate LLM-powered VPs, and the results are overall favorable. According to blinded human raters, VPs approached a “very good approximation of a real conversation” with “easily overlooked flaws,” and LLM-generated personalized feedback was nearly “on par with [feedback] from a trained human clinician-supervisor” (quoting operational criteria for rating=5, see Box S1 in Multimedia Appendix 1). Moreover, the VP demonstrably represented distinct patient preferences, including often expressing opinions that opposed clinician suggestions. LLM-as-clinician dialogues had authenticity ratings similar to human-as-clinician dialogues. LLM-generated ratings of feedback quality were similar to human ratings, whereas ratings of authenticity were much higher, which suggests inaccuracy. We also developed and validated instruments for rating dialogue authenticity, VP user experience, and feedback quality.

### Limitations

The most salient limitation is suboptimal reproducibility of human ratings. Importantly, the high between-replication variances suggest that inconsistencies could come from real differences in GPT performance in simulating the “same” case. Indeed, conversation creators noted significant differences in GPT responses on the second replication. High variances could also indicate within-rater idiosyncrasies and inconsistencies, and refined operational criteria and improved rater training could mitigate this. Low reproducibility could further arise from restriction of range: we asked GPT to provide excellent feedback, and for the most part it delivered. Soliciting a wider range of performance (eg, including intentionally substandard feedback) might reveal higher agreement. We noted difficulty in rating long conversations, especially when problems manifest in only a small part of an otherwise satisfactory conversation. It might help to rate shorter texts, which could be generated by splitting the text into chunks based on word count or using AI to extract salient subtexts. User experience was difficult to rate from a written transcript; we surmise that rating user experience as it dynamically unfolds in written text, or viewing a recorded performance, would be more meaningful. Importantly, our analyses adjusted for within-rater correlation, which helps mitigate rater inconsistencies for the purposes of this study.

GPT–generated ratings also had low reproducibility, but variance arose from run-to-run inconsistencies rather than replications. The data suggest that within a given analysis run, GPT assigns a similar rating level to all conversations; and on different runs it assigns different rating levels (ie, a different baseline). Providing training examples would likely improve consistency (ie, standardization).

There are other limitations. We adjusted the operational criteria for ratings between conversation creation and final ratings, thus precluding a meaningful evaluation of intrarater test-retest reliability. These VPs used only written text; however, authenticity was high even with this limitation. Moreover, we note that much clinical work now occurs using text communication. Recently released LLMs now support live bidirectional audio and video. We implemented just 2 topics from outpatient internal medicine and a limited spectrum of patient preferences; however, our approach easily extends to other topics and contextualizing features. Finally, for this intrinsic evaluation study, the clinician role was played by study investigators rather than real learners; real-world performance will be investigated in future extrinsic evaluations.

### Implications

We demonstrated proof of concept for scalable, globally accessible, and low-cost LLM-powered VPs. The unscripted, responsive dialogues contrast sharply with most existing VPs, for which authentic and flexible dialogue is notoriously difficult to replicate and often not attempted. Such authenticity will facilitate training, assessment, and research on shared decision-making [13-16] and other management reasoning processes [11,12,20]. Although patient preferences were not always perceivable, this parallels real life. A patient’s preferences will not surface in every patient-clinician encounter and often require elicitation by a skilled clinician [67]. Accordingly, the LLM’s ability to perceptibly represent preferences is commendable. Using this LLM-powered approach, thousands of preference-sensitive VPs can be created with much higher efficiency, and potentially higher authenticity, than current labor-intensive methods. A VP is “created” as a 1-page document, and permutations are incorporated by changing a few sentences. Such permutations (ie, preferences, comorbidities, social determinants of health, and system constraints) will prove invaluable in training and assessing contextualized care [17-19].

Our findings support the use of LLMs to deliver specific, actionable feedback to clinicians. This fills an important, long-recognized gap in clinical training [24-27]. Although LLM-generated feedback was not perfect, it was very good. If future research can improve feedback quality—perhaps using defined rubrics—it could support education across the continuum of clinician training and extending beyond VPs, including audio-recorded encounters involving simulated or real human patients and encompassing practicing physicians (eg, automated feedback on actual patient-clinician conversations for continuous professional development).

Subgroup comparisons clarify nuanced understanding. GPT-4.0-Turbo outperformed GPT-3.5-Turbo in both dialogs and feedback, albeit at substantially greater cost. By contrast, the absence of differences in all other comparisons of feedback is expected and thus reassuring (ie, we would not expect feedback quality to differ by topic or persona). LLM-as-clinician dialogues generated a less realistic user experience even though dialogue authenticity was similar. Dialogues for the poor medical student persona had low ratings; we attribute this to failure of the LLM to respond appropriately to poor performance (eg, by volunteering information or not expressing confusion) and raters’ perception that the student’s performance was unnatural.

We present evidence supporting the validity of scores from 3 instruments, rating dialogue authenticity, user experience, and feedback quality. Items were well grounded (ie, *content* evidence), and we confirmed expected *relations with other variables* (higher ratings for advanced LLM models and human clinician personas). Reproducibility (ie, *internal structure*) was suboptimal; however, our data suggest that inconsistencies arise, at least in part, from variation in LLM performance rather than rater idiosyncrasies. The data on features that detracted from or enhanced conversation quality provided evidence regarding investigators’ *response processes*, which largely align with the constructs embodied in the instrument items. We have suggested several steps that could improve reproducibility in future work.

Zero-shot LLM-generated ratings were suboptimal. LLM feedback ratings were similar to pair-matched human-generated ratings, but reproducibility was low. Dialogue ratings were higher than humans’ and presumably inaccurate, perhaps because GPT was rating itself. We speculate that a different LLM might be more objective. Providing examples (eg, few-shot learning) may also be needed. We had reservations that GPT could provide meaningful ratings of user experience (ie, an innately human perception) and thus did not attempt this. Future research could explore this.

Although LLMs are known to occasionally render biased responses, we did not detect any instances of bias in these conversations. We did encounter problems arising from rules built into GPT to *prevent* such responses: for example, when we tried to incorporate certain social determinants of health (such as race or income status), GPT would occasionally reject these as inappropriate—even though they were well-intentioned. We also built rules into our LLM prompt to identify and correct potentially biased statements from the clinician-user. We tested these during the prompt engineering phase, but not during formal conversation creation. We recommend ongoing attention to bias in future simulations.

Our findings suggest additional avenues for research. All these innovations—the LLM-powered VPs, LLM-generated feedback, LLM-clinician, and LLM-generated ratings—would benefit from further-refined prompt engineering and iterative evaluation. We also wonder if performance might be improved using fine-tuned LLMs with health care conversations as training data. As we found, LLMs respond differently every time; this is a strength (eg, spontaneous and natural dialogue), but also a liability (eg, inconsistent conditions for assessment or training). What are the consequences of such variability, and how can variability be mitigated when needed (such as for standardized assessment)? VPs could help address or inadvertently propagate bias and stereotypes; this warrants ongoing attention.

Finally, we note diverse potential applications of LLM-powered VPs, including clinical reasoning in other contexts (eg, inpatient and procedural settings), training nonclinicians (eg, nurses, therapists, pharmacists, and patients), education beyond clinical reasoning (ie, basic knowledge [through case-based learning], communication, teamwork, interprofessional education, tasks such as cognitive behavioral therapy or motivational interviewing, and socialization into the clinical role), and generating transcripts for research (eg, for studies comparing different feedback approaches). LLM-powered VPs could also help test clinical interventions (eg, novel workflows, informatics tools [software as a medical device], and AI innovations) or rehearse specific high-stakes scenarios (“digital twin”).

## Data Availability

The case descriptions used in this study are provided in the online supplemental materials; these can be used with ChatGPT or the OpenAI GPT application programming interface. The Python code was published previously [38]. The dataset of quality ratings is available from the corresponding author upon request within 12 months of publication.

## Appendix

Multimedia Appendix 1 Generative pretrained transformer (GPT) prompts, a representative conversation, details on instrument development and operational criteria for rating dialogue and feedback quality, planned case permutations, variance components for ratings, and ratings for specific study subgroups.

## Abbreviations

AI: artificial intelligence
API: application programming interface
GPT: generative pretrained transformer
ICC: intraclass correlation coefficient
LLM: large language model
VP: virtual patient
