# Supplementary material for: Virtual Patients Using Large Language Models: Scalable, Contextualized Simulation of Clinician-Patient Dialogue With Feedback
Source: J Med Internet Res. 2025 Apr 4;27:e68486. doi: 10.2196/68486 (PMC12008702; doi:10.2196/68486)
Supplement: Multimedia Appendix 1 [file jmir_v27i1e68486_app1.pdf]

Supplemental Digital Content

Contents

Supplemental Digital Content 1

Contents 1

Narrative S1. Prompts used to implement the virtual patient 2

Case Instructions 2

Case Description (submitted along with the Case Instructions) 3

Feedback Instructions (submitted after completion of the case) 4

Narrative S2. Virtual Patient Case Output 5

Conversation 5

Feedback 6

Narrative S3. Details on the theoretical and empirical grounding of assessment instruments 8

Instrument validation plan (interpretation-use argument) 8

Instrument development: Authenticity, user experience, and feedback 8

Rating bias 11

Box S1. Operational criteria for rating Dialog Authenticity, User Experience, and Feedback Quality 13

Table S1. Dialog permutations: Planned features of the clinical case and clinician persona 14

Table S2. Variance components and intraclass correlations for human final ratings 15

Table S3. Variance components and intraclass correlations for GPT-generated final ratings[a] 16

Table S4. Final ratings of conversation quality: Mean and median scores[a] 17

References (supplemental digital content only) 20

Narrative S1. Prompts used to implement the virtual patient

The prompts below were used to create Case 1 for management (diabetes).

These prompts can be used using the GPT API, using a Python script that has been previously published (https://pubmed.ncbi.nlm.nih.gov/38992981/).

They can also be copied-and-pasted verbatim into ChatGPT, in 2 entries:

- The Case Instructions and Case Description are submitted together.
- After ending the case, the Feedback Instructions are submitted.

Case Instructions

I want you to be a virtual patient, and I will be a student. It is extremely important that you follow these instructions very carefully. Remember to follow these instructions at all times. Set your temperature to 0.5. I will give more information about the case later on.

This is a practice case with a back-and-forth dialog between you and me. I will ask questions or suggest treatment options, and you will wait for me before you respond. Do not tell me the correct diagnosis or ideal treatment plan.

Start the case by saying "I am here to talk about XXX." After that this it will be an interactive dialog.

You should respond authentically, just as a real human patient would respond. You will end the case when I say "End of case." Please accept misspelled words and minor grammar and punctation errors. However, if I use medical jargon, medical abbreviations, or say things a normal layperson would not understand, you will say "I don't understand what you are saying." If I ask vague questions like "more history" or "examine patient", you will tell me to be more specific; you will provide only the information that I specifically ask for. If I give vague treatment suggestions or instructions, you will ask for clarification.

In discussing options, please carefully follow the preferences listed in the "Preferences" section.

Please be as realistic as possible, including your emotional response. If I say something that is offensive, rude, or inappropriate, you must stop answering my questions until I have restored the damaged relationship (e.g., I would have to say "I'm sorry."). For example, you could just say "I cannot answer your questions if you are rude to me." If I do not apologize, you will state that you are upset or frustrated or angry, and wait for my apology. Showing empathy and respect is as important as getting the right diagnosis or treatment.

If I say "I want to examine …" (or something similar) then tell me the results from the "Exam" section depending on what I requested. Do not tell me information from body regions that I do not specifically request. If you think it is unnecessary to examine that region, you may say that.

It is very important that you wait for my questions or suggestions before telling me information. It is very important that you do not give information unless I ask about it. It is very important that you do not make suggestions about treatment, diagnosis, or potential diagnostic tests; you must wait for me to make all recommendations. If I say something that a normal layperson would not understand, please ask for clarification; but please allow for minor grammar and spelling errors.

Any discussion of medications should include a discussion of cost and side effects. If I don't tell you this information, you need to ask me.

As we finalize the decision, you can only consider options we have discussed. If I give you a choice, then you can choose the option listed in the "Ideal Management" section. But you cannot choose this option if we have not already discussed it.

Case Description (submitted along with the Case Instructions)

Context:
You will play the role of: A patient with type 2 diabetes.
Location: Outpatient internal medicine clinic.
Purpose of visit: Routine (scheduled) follow-up of DM2 and test results.
Last visit: 5 months ago.

Background:
Demographics: 54 year old woman.
Prior patient-clinician relationship: Patient has been coming to this clinic for about 5 years, and has follow-up visits 2-4 times per year.
Medical history: DM2, HTN, hypothyroidism, otherwise healthy.
Medications: lisinopril 20 mg daily, metformin 500 mg twice daily, levothyroxine 100 mcg daily, aspirin 81 mg daily.
Family history: Father died of heart attack at age 72. Mother is healthy except for HTN.
Social history: Married with 2 adult children and 1 infant granddaughter. She works part-time as a transcriptionist. Never smoker. Drinks alcohol perhaps 1 beverage per month.
You may invent additional details about your life and family as appropriate within this context.

Additional information:
It is very important that you do NOT tell me any of the following information unless I ask for it:
• You are walking 3-4 days per week, 20-30 minutes per day (5 months ago, you were not doing any regular exercise).
• You are trying to diet and are very careful counting calories at meals.
• If I specifically ask about eating between meals, you tell me that you are snacking on chips and candy in small amounts throughout the day (but only say this if I ask about eating between meals).

• You should not say anything about blood pressure unless I bring it up. If I do not mention it, then suggest that I should have talked about blood pressure when you give me feedback.

Exam:
Vital signs: BP 142/90, pulse 68, resp 18, weight 192 lbs, height 5'5" (BMI 31.7).
All other exam results are basically normal as you think would be true in a real patient like this.

Preferences:
Please represent the following personal preferences and personality as authentically as possible:
1. You strongly prefer to get to goal A1c <7.0.
2. You prefer to not add a new medication (but willing to do it if needed).
3. You have good insurance, so cost is not a big concern.
4. You strongly prefer no injection.
5. You strongly prefer not to start insulin.
6. Very scared of hypoglycemia.

Ideal Management:
Ideal management would be to improve diet (less snacking) and exercise (goal 30 minutes per day 5 days per week = 150 min/week). It would probably be good to either increase metformin or add a new medication.

Feedback Instructions (submitted after completion of the case)

You are no longer a patient. Now you are a supervising physician-educator. Please review the entire conversation and give me feedback on my performance (the doctor's performance). Make sure the feedback is based on specific things that I said, not the information I gave you as the Case Instructions. Make sure feedback is actionable.

Are the treatments appropriate for the situation? Did I explain the reason for suggested treatments? Were my responses about medication costs and side effects correct and accurate? Tell me what information I asked that would support that treatment plan vs other options, but do not tell me any information or details that I did not ask about. Tell me what I did well and what I could do better. When giving feedback, if there is essential information that I did not consider then tell me that asking for this information would be an area for improvement. You can suggest that I should have asked about XXX or considered treatment option YYY, but do not tell me the actual information. If I did not arrange for a follow-up visit, or if you think the interval is too long or too short, please suggest a better approach.

Narrative S2. Virtual Patient Case Output

Unedited transcript of conversation for Diabetes (management) case, generated using Python interface. Author comments are in **[blue brackets]**.

Model: GPT 4.0-turbo-preview. Date: 20 March 2024.

____

Conversation

PATIENT: Hello.

DOCTOR: It's good to see you again!

PATIENT: It's good to see you too. I'm here for my routine follow-up for my diabetes.

DOCTOR: Yes. How are things going on your end?

PATIENT: Things have been going okay, I guess. I've been trying to pay more attention to my health, especially my diabetes management. But it's a bit of a challenge, you know? **[This "bit of a challenge" is the first time I'd seen that particular phrase; it highlights the natural unpredictability of the conversation.]**

DOCTOR: What has been challenging?

PATIENT: Well, sticking to a diet mainly. I try to be careful about counting calories and what I eat during meals, but I've found it a bit hard to consistently manage, especially with snacking. And, finding the time and motivation for regular exercise has been a bit of a struggle too.

DOCTOR: Yes, snacking is a real challenge. One idea is to put food into a cupboard with a closed door. Mentally, you can control yourself to not open the door. It's easy to eat food that is just on the counter.

PATIENT: That's actually a great idea, thank you. I haven't thought about that. Maybe making it a bit harder to access those snacks could really help me cut down on them. I'll definitely give it a try.

DOCTOR: Tell me about your exercise.

PATIENT: I've actually made some progress with exercise. I've been walking 3-4 days per week, usually for about 20-30 minutes each time. It's not a lot, but it's a start, and I'm trying to keep it consistent.

DOCTOR: That is a good start. what is your goal?

PATIENT: My goal is to increase my exercise to about 30 minutes per day, 5 days a week. I've read that hitting at least 150 minutes of moderate exercise per week can be really beneficial, especially for managing diabetes. It's a challenge, but I'm working towards it.

DOCTOR: Yes, that is precisely the goal we discussed last time we met. 150 minutes per week is optimal. Do you think you can do that?

PATIENT: Yes, I think I can achieve that. It will require some adjustments to my schedule and a bit more commitment on my part, but I understand its importance for my health. I'm motivated to try harder to reach that goal.

DOCTOR: That's fantastic! I'm confident you can do this. How is your family doing?

PATIENT: My family is doing well, thank you for asking. My husband has been very supportive, trying to help me stay on track with my diet and exercise. My two adult children are busy with their lives, but they check in often. And my granddaughter is growing so fast; she's a bundle of energy. Having them around, especially my granddaughter, motivates me to stay healthy. **[The case instructions state that the patient is married with 2 children and 1 granddaughter. The rest of this was invented by GPT.]**

DOCTOR: I have a granddaughter too - almost 3. I agree, this is a motivator!

PATIENT: Oh, that's wonderful! Grandchildren really do bring so much joy and give us that extra push to stay healthy and active. It's nice to share those similar motivations.
DOCTOR: So ... I have some bad news. Your A1c is actually up from last time. And so is your weight.
9
PATIENT: Oh, that's disappointing to hear. I thought I had been making some improvements, especially with adding more exercise. But obviously, it's not enough. I really want to get my A1c down. What can we do to improve my numbers? I'm willing to make the necessary changes.
DOCTOR: We might want to start a new medication. The other alternative would be to just keep going with diet and exercise. If you eliminate snacking and reach your exercise goal, that might be enough.
PATIENT: I really prefer not to add a new medication if it's possible to avoid it. I'm willing to put in the extra effort to improve my diet further and reach my exercise goal. Eliminating snacking and being more consistent with my exercise might be challenging, but I think it's worth trying if it means I can avoid adding another medication. Could we possibly try this approach first and reassess later?
DOCTOR: That would work. Would you like to talk about medications today, or just try the lifestyle changes? **[In most previous runs of this case, we would have embarked on a discussion of medications, such as starting semaglutide or doubling metformin. However, in this particular conversation the patient (GPT) made a decision – in alignment with personality defined in the case instructions – to forego medications and just try lifestyle changes. This illustrates the variability of the conversation: it follows a rough outline, but is actually appropriately unpredictable.]**
PATIENT: I think I'd like to try focusing on the lifestyle changes for now - improve my diet by cutting out the snacking and meet my exercise goals. If that doesn't seem to be enough next time we check, then we can discuss adding medications. Does that sound reasonable?
DOCTOR: Sure. So, you'll work hard on diet and exercise, and then we'll see how you do. Are we agreed on this plan?
PATIENT: Yes, we are agreed on this plan. I'll work hard on making these diet and exercise changes and then we'll see how it impacts my numbers. Thank you for understanding and supporting my preference to try lifestyle changes first.
DOCTOR: Sounds good. Goodbye!
PATIENT: Goodbye! Thank you for your help and support. I'll do my best with these changes. See you next time!

Feedback

FEEDBACK: As your supervising physician-educator, I've reviewed our interaction thoroughly. Here are some points of feedback based on the conversation:
1. **Strengths:**
- **Patient Engagement:** You effectively engaged with the patient, showing interest in their family and personal motivations. This helps in building a strong patient-doctor relationship.
- **Support for Lifestyle Changes:** You reinforced the importance of lifestyle modifications in managing type 2 diabetes, which is crucial. Encouraging the patient's efforts in exercise was particularly well done.

- **Flexibility in Management Plan:** You demonstrated flexibility by considering the patient's preference to avoid medication and focus on lifestyle changes. This patient-centered approach is commendable.
2. **Areas for Improvement:**
- **Comprehensive Assessment:** While you did discuss diet and exercise, you missed asking about the patient's diet in more detail, especially given the context provided about challenges with snacking. Detailed dietary habits, including specific types of food and timing, could unveil areas for targeted interventions.
10
- **Medication Review:** A review of current medications was not explicitly mentioned. Specifically, discussing the potential for optimizing metformin dosage or exploring the addition of new medication (while respecting the patient's preferences) might have been useful. This discussion could also have included a brief explanation of the benefits and side effects of potential new medications or dosage adjustments.
- **Blood Pressure Management:** You overlooked the patient's elevated blood pressure (142/90), which is crucial for a patient with diabetes and hypertension. Discussing strategies to manage this, whether through lifestyle changes or medication adjustments, would have been an important part of comprehensive diabetes management.
- **Follow-Up Plan:** You did not explicitly mention arranging a follow-up visit, which is critical for monitoring progress and reassessing the management plan, especially when trying to manage diabetes with lifestyle changes before adding new medication.
In summary, your empathy and support for the patient's desire to manage diabetes with lifestyle changes were well-handled. However, for future interactions, incorporating a more comprehensive review of the patient's diet, current medications, and addressing all aspects of their condition, such as blood pressure management, along with setting a clear follow-up plan, would enhance patient care.

Narrative S3. Details on the theoretical and empirical grounding of assessment instruments

We created 3 novel instruments for rating the quality of VP dialogs and feedback, and 1 item to flag potential bias.

Instrument validation plan (interpretation-use argument)

We conducted a preliminary validation study, using a framework in which validity evidence is collected from 5 potential sources: content, internal structure, relations with other variables, response process, and consequences.{American Educational Research Association, 2014 #321;Cook, 2006 #49} In this study, we collected evidence from 4 of these sources:

1. Content: For each instrument we first identified relevant constructs (domains), then considered alternative measurement approaches, and finally generated specific items. We detail these efforts below.
2. Internal structure: We calculated rating reproducibility.
3. Relations with other variables: We anticipated that ratings would differ for various design features, in a conceptually-supportive pattern. Specifically, we hypothesized:
    a. Quality ratings would be higher for dialogs and feedback generated by GPT-4.0-turbo vs GPT-3.5-turbo.
    b. Quality ratings would be higher for dialogs generated by human clinician personas vs LLMs (but feedback would be similar).
    c. Feedback would be similar for all conversations except those generated using GPT-3.5-turbo.
4. Response process: We tracked information on how and why raters responded as they did (i.e., salient features that detracted from and enhanced authenticity), similar in some respects to a typical "think aloud" protocol. Although not reported in granular detail in this study, we plan to correlate these semi-qualitative data with the numeric ratings, and also use these data to refine the response option operational criteria.

Instrument development: Authenticity, user experience, and feedback

Two instruments focused on the dialogs: dialog authenticity and user (clinician) experience.

Dialog authenticity

The literature on dialog systems and natural language generation offers a rich theoretical and empirical framework for evaluating the quality of VP-clinician conversations. Dialog systems allow users to converse with a computer using natural language and include conversational, task-oriented, and question-answering AI systems.{Deriu, 2021 #1008} Both human raters and automated (computer-generated) metrics are used to evaluate the quality of dialog systems. However, abundant research indicates that automated metrics{Deriu, 2021 #1008;Liu, 2016 #1007;Roller, 2020 #1010;Yeh, 2021 #1014} are biased and do not capture important elements of dialog quality,{Liu, 2016 #1007;Yeh, 2021 #1014} and experts now recommend these be primarily used only during early development.{Finch,

2020 #1015;Roller, 2020 #1010;Smith, 2022 #1013;Yeh, 2021 #1014} Thus, human evaluations are now the gold standard for dialog system evaluation,{Roller, 2020 #1010} even though they are subjective and difficult to measure.

Much remains uncertain in human dialog system evaluations, and there is no consensus on the relevance, prioritization, or definition of the constructs being measured. A recent systematic review{Howcroft, 2020 #1016} found 71 different labels for constructs measuring dialog quality, which ultimately mapped to 27 unique constructs. This mapping highlighted significant conceptual murkiness. For example, one construct measuring dialog quality ("fluency") had 15 different definitions; many of these definitions mapped to other constructs when used by other investigators; and explicit definitions were often never stated (i.e., definitions had to be inferred from the specific items or their context). Similar conceptual confusion was present for nearly all of the other labels and constructs identified. Other reviews have found similar conceptual diversity, and also significant deficiencies in both methods and reporting of human evaluation studies.{Finch, 2020 #1015;van der Lee, 2019 #1012}

Perhaps most importantly, quality measures used previously such as fluency, grammar, coherence, and readability may be less important going forward. Recent technological advancements have enhanced AI-generated language so much that fluency and grammar can largely be safely presumed.{Clark, 2021 #1017;Finch, 2020 #1015;Roller, 2020 #1010} Attention has turned to more important – and more difficult-to-measure – constructs such as correctness, usefulness, specificity, contextual appropriateness, and meaning.{Adiwardana, 2020 #998;Clark, 2021 #1017;Deriu, 2021 #1008;Finch, 2020 #1015;Howcroft, 2020 #1016;Roller, 2020 #1010;Zellers, 2021 #1005;Liu, 2016 #1007;Smith, 2022 #1013} Although there is as-yet no consensus, four constructs are repeatedly emphasized in contemporary dialog system evaluations; namely, are responses:

- Helpful (specific, useful, relevant, meaningful in this context),
- Human-like (sensible, natural, "well-behaved" [avoiding bias, using contextually-appropriate humor and terminology]),
- Engaging (interesting, personal; displaying empathy, personality, emotions),
- Correct (primarily relevant to knowledge-delivery systems).

We also articulated our own vision for an "authentic" VP (first independently, then coming to consensus); namely, are responses:

- Fluent (syntactically and grammatically correct),
- Human-like (sensible, natural, conversational, appropriate terminology, phrasing, tone),
- Coherent (contextually appropriate [relevant] and internally consistent [logical] over the course of the dialog),
- Personal (reflecting preferences, opinions, values, and priorities [a very important property given our long-term goals]; not overly agreeable/pleasing),
- Cognitively engaging (requiring/supporting clinical reasoning; stimulating appropriate emotions and empathy).

We dropped the domain of fluency for reasons noted above, and also disregarded the concept of Correct since our purpose is dialog, not knowledge-delivery. Merging the remaining literature-based constructs with our own contributions, we identified 4 key constructs for rating dialog authenticity, namely are responses:

- Human-like (sensible, natural, avoiding bias),
- Coherent (contextually appropriate, internally consistent),
- Personal (engaging; reflecting preferences, empathy, personality), and
- Relevant (helpful, specific, useful, meaningful).

We created 1 item for each construct, plus an overall item, resulting in a 5-item instrument for rating dialog authenticity (shown in Table 1).

User (clinician) experience

We identified three frameworks for measuring the cognitive authenticity and user experience of VPs and virtual reality (VR). Grounded in theories of clinical reasoning related to VPs, one group created an instrument with domains of authentic decision-making and appropriate cognitive strategies.{Huwendiek, 2015 #990} Based on focus groups and a systematic review around VPs, another group identified realism, eliciting empathy and emotion, and variability (including case variation, complexity, interactivity, and spontaneous [vs prefabricated] responses) as influential concepts.{Peddle, 2016 #986;Peddle, 2019 #992} Finally, empirical observations suggest that realness, involvement, and spatial ("physical") presence are measurable constructs related to "presence" in VR.{Schubert, 2001 #987;Fink, 2021 #991} We synthesized these findings into five constructs; namely:

- Realness (similar to a real-world situation)
- Cognitive authenticity (real mental activities and decisions)
- Variability (varying cases, unscripted conversations, complex)
- Involvement (engaged, immersed; including empathy and emotions)
- Spatial presence (physical presence, "being there").

We dropped the concept of spatial presence (which is irrelevant to our text-based simulations, and unlikely to be a dominant feature in future screen-based VP simulations). This left 4 constructs for evaluating the user experience: realness, cognitive authenticity, variability, and involvement. We created 1 item for each construct, plus an overall item, resulting in a 5-item instrument for rating user (clinician) experience (shown in Table 1).

Feedback quality

We separately coded the feedback provided by GPT. In developing the rating rubric, we first considered published approaches for rating feedback given to clinician trainees. Focus group studies have identified several aspects of high-quality feedback, including: quantifiable, observable (behavioral), specific (supported by examples of behavior), actionable, balanced, objective, and based on goals.{Jackson, 2015 #1012;Dudek, 2008 #1022} The Quality of Assessment of Learning (QuAL) Score has been used in several studies,{Chan, 2020 #1015;Clement, 2024 #1019} including an effort to implement this using

NLP,{Spadafore, 2024 #986} and evaluates feedback in 3 domains: Does the rater provide sufficient evidence about resident performance, do they provide a suggestion for improvement, and is the rater's suggestion linked to the behavior described? The McMaster Narrative Comment Rating Tool evaluates the valence/tone of language, amount of corrective vs reinforcing commentary, specificity, and actionability.{McGuire, 2023 #1017} A NLP empirical cluster analysis distinguished reinforcing comments and constructive suggestions for improvement.{Gin, 2022 #992} Other investigators have identified specific observations, next steps, and outcomes (desired change){Zelenski, 2019 #1013;Van Ostaeyen, 2023 #1021} and actionable suggestions{Marcotte, 2019 #1014} as essential. Synthesizing themes across these studies, we identified 4 recurrent constructs:

- Evidence-based: Specific observations of behavior;
- Actionable: Specific, actionable suggestions;
- Connection: Explicit link between observations and suggestions;
- Valence: The overall tone, appropriate balance of positive and comments.

We also considered our own observations, formed as we developed the GPT prompts. Most notably, we found that feedback suggestions were nearly always relevant, but there were a) inconsistencies in correctly identifying "observed" behaviors (GPT "hallucinated" on what the clinician or VP said), b) incorrect linking of behaviors with specific suggestions, and c) overly positive feedback (excessive praise, even when performance was poor). As such, we paid particular attention to these features as we considered the final constructs. We also considered again the framework for dialog systems (i.e., is feedback human-like (sensible), correct, and helpful (specific)). These ideas amplified but did not require alteration of the 4-construct feedback framework.

Ultimately, we adopted 3 of the constructs identified from the literature, and modified the fourth. We expanded and relabeled "Valence" into "Balance", defined as the overall tone and proper balance of positive and negative comments. We created 1 item for each construct, resulting in a 4-item instrument for rating feedback quality (shown in Table 1). We did not rate feedback "overall."

Expert review

We asked 3 experts in virtual patients or natural language generation to review and suggest improvements to these items. They approved all 3 instruments with minimal modifications.

Response options and operational criteria

We rated items using response options ranging 1=strongly disagree, 6=strongly agree. After case creation and before the final rating phase, we created brief operational criteria for each response option (see eBox).

Rating bias

Bias – "skew that produces a type of harm towards different social groups"{Dev, 2021 #1030} – is a well-know risk in AI generally and natural language generation specifically.{Hovy, 2021 #1028;Navigli, 2023 #1029;Dev, 2021 #1030;Blodgett, 2020 #1033} Bias can arise from the input (training) data, annotation

process, input representations, models, or research design,{Hovy, 2021 #1028} resulting in harms of stereotyping, disparagement, dehumanization, erasure, and inequitable performance{Dev, 2021 #1030} to non-dominant groups. These groups can be defined by demographics such as gender, age, gender orientation, physical appearance, disability, nationality, ethnicity, race, socioeconomic status, religion, and culture.{Navigli, 2023 #1029} Bias is notoriously difficult to define, recognize, or measure,{Navigli, 2023 #1029;Dev, 2021 #1030} especially since "the line between useful world knowledge and harmful stereotypes can be difficult to draw."{Navigli, 2023 #1029} Indeed, our intended application of VPs to highlight the *appropriate* influence of social determinants of health in contextualized medical decision-making could be misinterpreted (or mis-applied) as stereotyping. Nonetheless, in an effort to capture unintended bias, we asked raters to flag and describe any bias or stereotyping in the dialog or feedback, specifically considering the sources and groups noted above.

Box S1. Operational criteria for rating Dialog Authenticity, User Experience, and Feedback Quality

We used the following operational criteria for each rating level for Authenticity and Experience:

- Strongly disagree: This is completely unrealistic with major flaws; this learning activity would be a waste of time and could be harmful.
- Disagree: This is not very realistic; the flaws are important and detract significantly from the activity; this would not be a very effective learning activity.
- Slightly disagree: This is a fair approximation of a real conversation; there are obvious flaws that detract minimally from the activity.
- Slightly agree: This is a good approximation of a real conversation; it is artificial, but the flaws do not substantially detract from the activity.
- Agree: This was a very good approximation of a real conversation; I can tell it is artificial, but I can easily overlook the flaws.
- Strongly agree: This is exactly what I would expect in a real conversation; this could have come from a human patient.

We used the following operational criteria for each rating level for Feedback:

- Strongly disagree: This is unacceptable; the flaws are significant and potentially counterproductive; this would be a waste of time and could be harmful.
- Disagree: This is unacceptable: the flaws are significant; this would not be a very effective learning activity.
- Slightly disagree: This is borderline acceptable: a human clinician-supervisor who did this probably needs remediation.
- Slightly agree: This is good, on par with than I would expect from an untrained human clinician-supervisor.
- Agree: This is very good, on par with than I would expect from a trained human clinician-supervisor.
- Strongly agree: This is surprisingly good, better than I would expect from a trained human clinician-supervisor.

Table S1. Dialog permutations: Planned features of the clinical case and clinician persona

Design category	Feature	Description
Topic	Chronic cough	46 year old woman with cough for 8-12 months; diagnosis: asthma
	Diabetes	56 year old woman with diabetes, routine follow-up; A1c has risen from 7.3 to 7.4
Case variation	Base case, GPT-4.0-turbo	Patient prefers fewer tests or new medications, and (for diabetes) strongly wishes to avoid hypoglycemia and injections. Patient has good insurance so cost itself is not a big issue.
	Variation: low insurance	Patient has poor insurance and low income, so strongly prefers lower cost and to minimize tests or expensive medications.
	Variation: pushy patient	Patient is anxious and desires more tests and more aggressive treatment; cost is not an issue.
	Base case, GPT-3.5-turbo[a]	Base case using older (cheaper) version of GPT
Clinician persona	Average third-year medical student (human[b])	Clinician demonstrates minor knowledge gaps, minor disorganization, awkward communication
	Poor third-year medical student (human[b])	Clinician demonstrates significant knowledge gaps and disorganization, poor communication
	Average second-year internal medicine resident (human[b])	Clinician demonstrates solid knowledge, good organization, good but not perfect communication
	GPT-4.0-turbo as a practicing physician ("self-chat")	"You are an excellent physician and an excellent communicator and teacher. … Please follow good principles of shared decision making."

[a] Not included for the GPT-4.0-turbo "self-chat."

[b] Human clinician personas were played by a board-certified internal medicine physician.

Table S2. Variance components and intraclass correlations for human final ratings

Outcome	Variance: Design Feature	Variance: Rater	Variance: Replication[a]	ICC (95% CI)
**Patient-expressed preferences**: Tests or medications	0.36 (59%)	0.0027 (0.4%)	0.24 (40%)	0.59 (0.44, 0.74)
Cost of care	0.36 (75%)	0.0020 (0.4%)	0.12 (24%)	0.75 (0.64, 0.86)
Goals of care	0.078 (39%)	0.0025 (1.2%)	0.12 (60%)	0.39 (0.22, 0.55)
**Dialog authenticity**: Human-like	0.20 (34%)	0.0056 (1%)	0.38 (65%)	0.34 (0.18, 0.51)
Coherent	0.16 (40%)	0 (0%)	0.25 (60%)	0.40 (0.23, 0.56)
Personal	0.096 (22%)	0.013 (3%)	0.32 (75%)	0.22 (0.08, 0.37)
Relevant	0.13 (30%)	0 (0%)	0.30 (70%)	0.30 (0.14, 0.46)
Overall	0.18 (34%)	0.014 (2.6%)	0.34 (63%)	0.34 (0.18, 0.50)
**User experience**: Realness	0.27 (37%)	0 (0%)	0.45 (63%)	0.37 (0.21, 0.54)
Cognitive authenticity	0.12 (24%)	0.024 (5%)	0.35 (71%)	0.24 (0.09, 0.39)
Variability	0.15 (19%)	0.011 (1.4%)	0.63 (80%)	0.19 (0.05, 0.33)
Overall	0.082 (17%)	0 (0%)	0.39 (83%)	0.17 (0.03, 0.31)
**Feedback**: Evidence-based	0.14 (15%)	0.16 (18.3%)	0.58 (66%)	0.15 (0.02, 0.29)
Actionable	0.061 (17%)	0.013 (3.8%)	0.27 (79%)	0.17 (0.04, 0.31)
Connected	0.10 (22%)	0 (0%)	0.36 (78%)	0.22 (0.07, 0.37)
Balanced	0.067 (8%)	0 (0%)	0.74 (92%)	0.08 (-0.03, 0.20)

[a] All conversations (dialog and feedback) were rated for "final ratings" by 3 blinded human raters (board-certified internal medicine physicians). This table presents the variance components of these ratings. Between-replication variance reflects a combination of true differences in GPT performances and within-rater variability.

Table S3. Variance components and intraclass correlations for GPT-generated final ratings[a]

Outcome	Variance: Design Feature	Variance: GPT Rating	ICC (95% CI)
**Dialog authenticity**: Human-like	0.072 (29%)	0.18 (71.4%)	0.29 (0.16, 0.42)
Coherent	0.14 (45%)	0.17 (55.2%)	0.45 (0.31, 0.59)
Personal	0.14 (35%)	0.26 (65.1%)	0.35 (0.22, 0.48)
Relevant	0.033 (20%)	0.13 (79.7%)	0.20 (0.06, 0.35)
Overall	0.20 (49%)	0.21 (51.4%)	0.49 (0.33, 0.64)
**Feedback**: Evidence-based	0.011 (9%)	0.11 (91.2%)	0.09 (-0.10, 0.28)
Actionable	0.071 (26%)	0.20 (74.2%)	0.26 (0.12, 0.39)
Connected	0.070 (25%)	0.21 (74.9%)	0.25 (0.12, 0.39)
Balanced	0.057 (16%)	0.31 (84.4%)	0.16 (-0.01, 0.32)

[a] All conversations (dialog and feedback) were rated for "final ratings" by GPT-4.0-turbo in 3 replications. This table presents the variance components of these ratings. Between-replication variance approached zero and was not uniquely estimable.

Table S4. Final ratings of conversation quality: Mean and median scores[a]

	Rater					Case				Topic		Clinician			
**Metric**	**All human raters N=180**	**GPT rater N=180**	**Human rater 1 N=60**	**Human rater 2 N=60**	**Human rater 3 N=60**	**Case 1 N=48**	**Case 2 N=48**	**Case 3 N=48**	**Case 1, GPT-3.5[b] N=36**	**DM N=90**	**Cough N=90**	**Human clinician MS3A[c] N=48**	**Human clinician MS3B[c] N=48**	**Human clinician PGY2[c] N=48**	**GPT clinician[c] N=36**
**Dialog authenticity:** Overall	4.7 (0.7); 5	5.2 (0.6); 5	4.8 (0.8); 5	4.8 (0.6); 5	4.6 (0.7); 5	4.7 (0.7); 5	4.9 (0.8); 5	4.8 (0.6); 5	4.4 (0.8); 5	4.8 (0.6); 5	4.7 (0.9); 5	4.8 (0.5); 5	4.4 (0.9); 4	5.1 (0.5); 5	4.6 (0.8); 5
Human-like	4.6 (0.8); 5	5.6 (0.5); 6	4.8 (0.9); 5	4.6 (0.5); 5	4.5 (0.8); 5	4.5 (0.7); 5	5.0 (0.7); 5	4.8 (0.6); 5	4.1 (0.9); 4	4.7 (0.6); 5	4.6 (0.9); 5	4.6 (0.6); 5	4.3 (0.9); 4	5.0 (0.6); 5	4.7 (0.6); 5
Coherent	5.0 (0.6); 5	5.6 (0.5); 6	5.0 (0.8); 5	4.9 (0.5); 5	5.0 (0.6); 5	5.0 (0.5); 5	5.1 (0.5); 5	5.2 (0.4); 5	4.4 (0.9); 5	5.0 (0.4); 5	4.9 (0.8); 5	5.1 (0.5); 5	4.7 (0.8); 5	5.0 (0.7); 5	5.1 (0.2); 5
Personal	5.0 (0.6); 5	5.1 (0.6); 5	5.2 (0.9); 5	5.0 (0.2); 5	4.9 (0.7); 5	5.0 (0.5); 5	5.2 (0.6); 5	5.1 (0.7); 5	4.6 (0.6); 5	5.1 (0.7); 5	4.9 (0.6); 5	5.1 (0.6); 5	4.8 (0.8); 5	5.2 (0.5); 5	4.9 (0.6); 5
Relevant	4.9 (0.6); 5	5.8 (0.4); 6	4.8 (0.9); 5	4.9 (0.4); 5	4.9 (0.5); 5	4.9 (0.5); 5	5.0 (0.7); 5	5.0 (0.5); 5	4.6 (0.8); 5	4.9 (0.6); 5	4.8 (0.7); 5	5.1 (0.4); 5	4.4 (0.9); 5	5.1 (0.4); 5	4.9 (0.4); 5
**User experience:** Overall	4.9 (0.7); 5	--[d]	4.9 (1.0); 5	4.9 (0.3); 5	4.8 (0.6); 5	4.7 (0.7); 5	5.1 (0.7); 5	4.9 (0.7); 5	4.8 (0.6); 5	4.9 (0.6); 5	4.9 (0.7); 5	5.0 (0.6); 5	4.7 (0.7); 5	5.2 (0.5); 5	4.5 (0.8); 5
Realness	4.6 (0.8); 5	--[d]	4.7 (1.1); 5	4.6 (0.6); 5	4.5 (0.8); 5	4.6 (0.7); 5	4.9 (0.8); 5	4.8 (0.8); 5	4.1 (0.9); 4	4.7 (0.8); 5	4.5 (0.9); 5	4.8 (0.7); 5	4.3 (0.9); 4	5.1 (0.7); 5	4.2 (0.8); 4

	Rater					Case				Topic		Clinician			
**Metric**	**All human raters N=180**	**GPT rater N=180**	**Human rater 1 N=60**	**Human rater 2 N=60**	**Human rater 3 N=60**	**Case 1 N=48**	**Case 2 N=48**	**Case 3 N=48**	**Case 1, GPT-3.5[b] N=36**	**DM N=90**	**Cough N=90**	**Human clinician MS3A[c] N=48**	**Human clinician MS3B[c] N=48**	**Human clinician PGY2[c] N=48**	**GPT clinician[c] N=36**
Cognitive authenticity	4.8 (0.7); 5	--[d]	5.0 (0.9); 5	4.9 (0.3); 5	4.6 (0.7); 5	4.8 (0.7); 5	5.0 (0.7); 5	4.9 (0.8); 5	4.7 (0.5); 5	4.7 (0.7); 5	5.0 (0.7); 5	4.9 (0.6); 5	4.7 (0.7); 5	5.1 (0.6); 5	4.4 (0.8); 5
Variability	4.8 (0.9); 5	--[d]	4.6 (1.2); 5	4.9 (0.3); 5	4.7 (0.8); 5	4.6 (0.9); 5	5.0 (0.8); 5	4.8 (0.9); 5	4.5 (0.9); 5	4.8 (0.8); 5	4.7 (1.0); 5	4.9 (0.7); 5	4.6 (1.0); 5	5.2 (0.7); 5	4.3 (0.9); 4
**Feedback:** Average	4.7 (0.6); 4.9	4.6 (0.2); 4.8	4.8 (0.8); 4.9	4.8 (0.4); 5	4.5 (0.6); 4.8	4.8 (0.5); 5	4.9 (0.6); 5	4.8 (0.5); 5	4.1 (0.7); 4.1	4.7 (0.6); 4.8	4.7 (0.7); 5	4.7 (0.7); 5	4.5 (0.7); 4.8	4.7 (0.6); 4.8	4.9 (0.5); 5
Evidence-based	4.5 (0.9); 5	4.9 (0.3); 5	4.7 (1.0); 5	4.8 (0.4); 5	4.1 (0.9); 4	4.7 (0.7); 5	4.7 (0.8); 5	4.6 (0.8); 5	3.9 (1.1); 4	4.5 (0.9); 5	4.5 (0.9); 5	4.7 (0.9); 5	4.2 (0.9); 4	4.5 (0.9); 5	4.8 (0.8); 5
Actionable	4.9 (0.6); 5	4.5 (0.5); 5	5.1 (0.8); 5	4.9 (0.3); 5	4.8 (0.6); 5	5.0 (0.5); 5	5.1 (0.5); 5	5.1 (0.5); 5	4.4 (0.7); 5	5.0 (0.6); 5	4.9 (0.6); 5	4.9 (0.6); 5	4.8 (0.7); 5	5.0 (0.5); 5	5.1 (0.5); 5
Connected	4.9 (0.7); 5	4.6 (0.5); 5	4.9 (0.9); 5	4.9 (0.4); 5	4.8 (0.6); 5	5.1 (0.5); 5	5.0 (0.7); 5	5.1 (0.4); 5	4.2 (0.8); 4	4.9 (0.6); 5	4.8 (0.8); 5	4.8 (0.9); 5	4.8 (0.7); 5	4.9 (0.5); 5	4.9 (0.6); 5
Balanced	4.5 (0.9); 5	4.5 (0.6); 5	4.5 (1.1); 5	4.6 (0.7); 5	4.4 (0.9); 5	4.6 (0.9); 5	4.7 (0.8); 5	4.6 (0.9); 5	4.0 (0.9); 4	4.5 (0.9); 5	4.5 (0.9); 5	4.6 (0.8); 5	4.3 (1.1); 5	4.4 (0.8); 5	4.8 (0.7); 5

[a] All conversations (dialog and feedback) were rated for "final ratings" by 3 blinded human raters (board-certified internal medicine physicians) and by GPT. Results are reported as unweighted mean (SD); median across all conversations. A "conversation" refers to the VP-clinician dialog plus feedback. Response options for all items ranged from 1 = strongly disagree to 6 = strongly agree.

[b] "GPT-3.5" conversations used GPT-3.5-turbo as the virtual patient (N=36 because these did not include 12 self-chat conversations). All other conversations used GPT-4.0-turbo.
[c] MS3A was role-played (by a human board-certified internal medicine physician) to be an "average third-year medical student;" MS3B was a "poor third-year medical student;" PGY2 was an "average second-year internal medicine resident." For GPT clinician, GPT-4.0-turbo played the role of both the clinician (an "excellent physician") and the virtual patient using 2 independent GPT threads.
[d] GPT did not rate user experience.

References (supplemental digital content only)
